# A Multilocus Sequence Analysis Scheme for Phylogeny of *Thioclava* Bacteria and Proposal of Two Novel Species

**DOI:** 10.3389/fmicb.2017.01321

**Published:** 2017-07-13

**Authors:** Yang Liu, Qiliang Lai, Zongze Shao

**Affiliations:** ^1^School of Municipal and Environmental Engineering, Harbin Institute of Technology Harbin, China; ^2^State Key Laboratory Breeding Base of Marine Genetic Resources, Key Laboratory of Marine Genetic Resources, Third Institute of Oceanography, State Oceanic Administration, Collaborative Innovation Center for Exploitation and Utilization of Marine Biological Resources, Key Laboratory of Marine Genetic Resources of Fujian Province Xiamen, China

**Keywords:** *Thioclava*, MLSA, genome, dDDH, polyphasic taxonomy

## Abstract

A multilocus sequence analysis (MLSA) was established and performed on the genus *Thioclava*, including 23 strains isolated from diverse marine environments, with the aim of better differentiation of strains and species within this genus. The study was based on sequences of 16S rRNA gene and five protein-coding housekeeping genes, *gyrB, rpoD, dnaK, trpB*, and *recA*. In contrast to 16S rRNA gene-based tree that was unable to separate some species within this genus, each tree based on a single housekeeping gene and MLSA had consistently defined seven clades, corresponding to the five established ones and two novel ones. The digital DNA-DNA hybridization and average nucleotide identity analyses based on genome sequences of the representative strains reconfirmed the validity of the MLSA analysis, and recommended a 97.3% MLSA similarity as the soft species threshold and nine species representing the five known and four putative novel species. Two of the four new species were identified as *Thioclava sediminum* sp. nov. (type strain TAW-CT134^T^ = MCCC 1A10143^T^ = LMG 29615^T^) and *Thioclava marinus* sp. nov. (type strain 11.10-0-13^T^ = MCCC 1A03502^T^ = LMG 29618^T^) by using a polyphasic taxonomic approach. Taken together, the newly established MLSA in this study first described the variability and phylogeny of the genus *Thioclava* which contributes to better understanding its ecology and evolution.

## Introduction

*Thioclava* is a genus consisting of Gram-staining-negative, moderately halophilic and facultatively aerobic bacteria belonging to the family *Rhodobacteraceae* within the class *Alphaproteobacteria* (Sorokin et al., 2005). At the time of writing, the genus *Thioclava* consisted of the five published species, including the type species *Thioclava pacifica* (Sorokin et al., 2005), along with *Thioclava dalianensis* (Zhang et al., 2013), *Thioclava atlantica* (Lai et al., 2014a), *Thioclava indica* (Liu et al., 2015a), and the newly described *Thioclava nitratireducens* (Liu et al., 2017b). Until now, a large number of isolates of this genus were obtained from diverse marine environments and preliminarily identified by 16S rRNA gene sequencing. Some strains were found to capable of sulfur oxidation (Sorokin et al., 2005), degradation of crude oil (Wang et al., 2014), decomposition of dibenzothiophene (unpublished data), fixation of carbon dioxide (unpublished data), and power generation as electrochemically active bacteria (Rowe et al., 2015). Therefore, we speculate that the *Thioclava* bacteria may play a crucial role in the biogeochemical cycles of sulfur and carbon.

PCR-based methods combined with DNA sequencing have been widely applied in phylogenetic analyses of bacteria, especially for some groups that cannot be distinguished by conventional methods. The 16S rRNA gene is the most popular genetic marker for taxonomic identification of Bacteria and Archaea (Yarza et al., 2014). However, 16S rRNA gene sequence is unable to distinguish certain closely related strains and even species owing to the relatively high conservation, the heterogeneity of multiple copies, and horizontal gene transfer (HGT) in previously published studies (Kitahara and Miyazaki, 2013; Liu et al., 2013, 2015b; Tian et al., 2015). Similarly, almost all of *Thioclava* bacteria from our group and GenBank database could not be well classified with respect to the relatively high similarities of their 16S rRNA gene sequences (above 97%).

Since 2005, multilocus sequence analysis (MLSA) has become a powerful tool and has been performed frequently to elucidate taxonomic relationship and phylogenetic analysis of closely related strains (Gevers et al., 2005; Vinuesa, 2010; Peeters et al., 2016; Whatmore et al., 2016). In general, several housekeeping genes are chosen preferentially in MLSA schemes, such as *gyrB* (DNA gyrase, beta subunit), *rpoD* (RNA polymerase, σ^70^ factor), *dnaK* (Chaperone protein DnaK), *trpB* (tryptophan synthase, beta subunit), and *recA* (recombinase A), because they encode core metabolic enzymes (Charlebois and Doolittle, 2004); and more importantly, they own much higher discrimination than 16S rRNA gene (Glaeser and Kämpfer, 2015). Furthermore, the concatenated housekeeping genes could minimize the weight of HGT (Macheras et al., 2011) and/or recombination (Timilsina et al., 2015) and then provide accurate taxonomic position for closely related species and strains (Glaeser and Kämpfer, 2015). In addition, digital DNA-DNA hybridization (dDDH) and average nucleotide identity (ANI) analyses based on genome sequences of bacteria are rapid and reliable methods for identification of closely related strains (Konstantinidis and Tiedje, 2005; Meier-Kolthoff et al., 2013; Garrido-Sanz et al., 2016). In recent years, these approaches have been successfully used for numerous bacteria, e. g. the *Bacillus cereus* group (Liu et al., 2015b, 2017a), *Thalassospira* (Lai et al., 2014c), *Hyphomonas* (Li et al., 2014), *Burkholderia* (Peeters et al., 2016), but without considering members of the genus *Thioclava* to date. Consequently, whether these approaches can be used for the *Thioclava* bacteria remains to be further investigated.

In recent years, multiple strains of bacteria assigned to the genus *Thioclava* have been isolated from various marine environments, suggesting a critical role of this genus in distinct habitats. This study proposes MLSA analyses to resolve the taxonomic affiliation of novel strains and species within this genus. In this study, we created the new MLSA scheme based on five housekeeping genes with *gyrB, rpoD, recA, trpB*, and *dnaK* as well as 16S rRNA gene to resolve the taxonomic affiliation of a collection of the *Thioclava* bacteria isolated from various marine environments, and further verified by dDDH and ANI analyses. Four putative novel species were discovered and two of them were subjected to a polyphasic taxonomic characterization.

## Materials and methods

### Strains and culture conditions

Many *Thioclava* strains were isolated in recent years by our group, the representatives of which have been deposited in the Marine Culture Collection of China (MCCC). In this report, 23 strains were chosen for phylogenetic study (Table 1), among which, five were type strains, *T. pacifica* MCCC 1A06460^T^, *T. dalianensis* MCCC 1A03957^T^, *T. atlantica* MCCC 1A02612^T^, *T. indica* MCCC 1A00513^T^, and *T. nitratireducens* MCCC 1A07302^T^. As listed in Table 1, all strains were isolated from marine environments; 15 strains were isolated from coastal areas of China, such as Beihai Sea, Dalian Bay, Yellow Sea; two from the Pacific Ocean; one from the Indian Ocean; one from the Atlantic Ocean; and two from the Bering Sea. Other two strains, *Thioclava* sp. ES.031 and *Thioclava* sp. ES.031, were isolated from the Monterey Bay near Monterey city in California on the basis of the annotated information from IMG database (http://genome.jgi.doe.gov/). Unless otherwise specified, strains were grown aerobically on marine agar 2216 and in marine broth 2216 (BD, Difco) for 48 h at 28°C.

**Table 1:** The detailed information for all strains used in this study.

**MCCC No**.	**Original No**.	**Geographic origin**	**Isolated source**	**Coordinates**	**Clade**	**Species**
MCCC 1A00513^T^	DT23-4^T^	Indian Ocean	Seawater	34.67° N, 23.72° E	7	*T. indica*
MCCC 1A02612^T^	13D2W-2^T^	Atlantic Ocean	Sediment	26.02° S, 13.86° W	5	*T. atlantica*
MCCC 1A02765	IC9	Yellow Sea, China	Seawater	35.83° N, 120.80° E	2	*T. nitratireducens*
MCCC 1A02808	F1Mire-8	Beihai Sea, China	Sediment	20.90° N, 108.98° E	2	*T. nitratireducens*
MCCC 1A02813	F28-4	Beihai Sea, China	Sediment	21.57° N, 108.58° E	1	PNS 1
MCCC 1A02837	F36-6	Beihai Sea, China	Sediment	21.52° N, 108.67° E	1	PNS 1
MCCC 1A02857	F42-5	Beihai Sea, China	Sediment	21.43° N, 109.72° E	2	*T. nitratireducens*
MCCC 1A02959	JM3	Coastal area, China	Seawater	36.00° N, 120.00° E	2	*T. nitratireducens*
MCCC 1A03188	F36-7	Beihai Sea, China	Sediment	21.52° N, 108.67° E	1	PNS 1
MCCC 1A03190	F34-6	Beihai Sea, China	Sediment	21.48° N, 108.48° E	2	*T. nitratireducens*
MCCC 1A03502^T^	11.10-0-13	Yellow Sea, China	Seawater	No data	4	PNS 2
MCCC 1A03505	SRB-64	Coastal area, China	Seawater	27.22° N, 121.37° E	4	PNS 2
MCCC 1A03506	11.6-2-6	Coastal area, China	Seawater	27.22° N, 121.37° E	4	PNS 2
MCCC 1A03957^T^	DLFJ1-1^T^	Dalian Bay, China	Seawater	38.95° N, 121.90° E	6	*T. dalianensis*
MCCC 1A03973	DLFJ4-1	Dalian Bay, China	Seawater	39.02° N, 121.78° E	1	PNS 1
MCCC 1A03974	DLFJ5-1	Dalian Bay, China	Seawater	38.97° N, 121.77° E	1	PNS 1
MCCC 1A06460^T^	TL 2^T^	Pacific Ocean	Seawater	No data	3	*T. pacifica*
MCCC 1A07302^T^	25B10_4^T^	Bering Sea	Seawater	59.69° N, 179.34° E	2	*T. nitratireducens*
MCCC 1A07323	25B07-5-2	Bering Sea	Seawater	58.00° N, 176.20° E	5	*T. atlantica*
MCCC 1A08421	L04-15	Pacific Ocean	Seawater	3.10° S, 102.55° W	4	PNS 4
MCCC 1A10143^T^	TAW-CT134	Coastal area, China	Sediment	24.65° N, 118.17° E	1	PNS 1
	ES.031	Monterey Bay, USA	Intertidal Microbial Mat	36.81° N, 121.79° W	1	PNS 2
	ES.032	Monterey Bay, USA	Intertidal Microbial Mat	36.81° N, 121.79° W	1	PNS 3

T *Type strains; PNS, Putative new species*.

### DNA preparation, gene selection, PCR amplification, and sequencing

Bacterial genomic DNAs for the strains were extracted using the SBS extraction kit (SBS Genetech Co., Ltd. in Shanghai, China) following the manufacturer's instructions. In this study, the above-mentioned five housekeeping genes were chosen on the basis of the following criteria: (1) the common presence in all strains belonged to the core genes; (2) single copy; (3) a relatively high discrimination power relative to 16S rRNA gene; (4) no or little HGT and recombination events; (5) the same or similar evolutionary rate. PCR amplification and sequencing of 16S rRNA and *gyrB* gene were performed using the primers as described previously (Lai et al., 2014c), and included in Table S1. The four pair specific primers for other genes, *rpoD, recA, trpB*, and *dnaK*, were designed from the available genome sequences of five type strains in GenBank database and two in IMG database using the software Primer Premier version 5.0, which were marked, respectively in Table S1. These genes were amplified under nearly identical conditions. The 50 μL reaction mixtures contained 37 μL of double distilled water, 5 μL of 10 × Ex Taq buffer (Mg^2+^ Plus), 4 μL of dNTP mixture (10 mM), 1 μL of each primer (20 μM), 1 μL of genomic DNA template (10–30 ng/μL), 1 μL of Ex Taq™ DNA polymerase (TaKaRa, 5 U/μL). PCR amplifications were performed according to the following parameters in a Biometra T-Professional thermocycler (Biometra; Goettingen, Germany): initial denaturation at 94°C for 5 min; then 30 cycles of 30 s of denaturation at 94°C, 30 s of annealing at 55°C, and 1.0–1.5 min of extension at 72°C; and a final extension at 72°C for 10 min. More detail information was listed in Table S1. After amplification, PCR products and their concentration were verified by electrophoresis of 3 μL products on a 1% agarose gel and stained with ethidium bromide. A DL2000 marker (TaKaRa, China) was included to estimate the length of the amplification products. And then, the PCR products were purified using the PCR purification kit (Axygen Scientific, Inc., USA) according to the manufacturer's instructions. Finally, the purified products were sequenced with the ABI3730xl platform (Shanghai Majorbio Bio-Pharm Technology Co., Ltd., Shanghai, China) using the corresponding sequencing primers (Table S1).

The ambiguous bases and/or gaps of sequences for all genes of 16 strains were modified artificially on the basis of the height of the original sequencing peak using the software DNAMAN version 7.0 (Lynnon Biosoft, Quebec, Canada). The gene sequences of other seven strains were obtained from their genome sequences using a local BLAST function implemented in the software BioEdit version 7.0.9.0 (http://www.mbio.ncsu.edu/BioEdit/bioedit.html). All sequences were submitted to GenBank database. The accession numbers were assigned and listed in Table S2.

### Analysis of nucleotide diversity

All gene sequences were aligned using the software MAFFT version 7.311 under the default settings (Katoh and Standley, 2013) for nucleotide diversity analysis. Diversity indices of 16S rRNA gene, single housekeeping gene, and concatenated housekeeping genes sequences, such as numbers of allele and parsimony informative sites, mean G+C content (mol%), and *Ka*/*Ks* ratios (*Ka*: the number of non-synonymous substitutions per non-synonymous site, *Ks*: the number of synonymous substitutions per synonymous site), were analyzed using the software DnaSP version 5.10 (http://www.ub.edu/dnasp/). The ranges of similarities and mean similarities of single gene and concatenated genes sequences were analyzed using DNAMAN 7.0 under default parameters.

### Phylogenetic analysis

Prior to the phylogenetic analysis, all genes were subjected to an examination of sequence substitution saturation and recombination events. A substitution saturation assessment was performed using the software DAMBE version 5.3.10 (Xia, 2013). Putative recombination events were detected using the software RDP version 4.16 (Martin et al., 2015). This software was used for searching the occurrence of recombination events when different parts of the sequence data result in discordant topologies using different algorithms, RDP, Bootscan, MaxChi, Chimera, Geneconv, and SiScan. Statistical significance was set at *P* ≤ 0.01.

The five housekeeping gene sequences were imported into the software MEGA version 5.05 (Tamura et al., 2011) for alignment by amino acid. Once the open reading frame was determined, sequences were translated to amino acid and then aligned by Muscle (Codons) (Edgar, 2004) implemented in MEGA 5.05. The amino acid alignment was then back-translated to nucleotides, retaining the codon positions. The 16S rRNA gene sequences were aligned using MAFFT 7.311. The appropriate nucleotide substitution models for gene(s) were determined using the software jModelTest version 2.1.7 (Darriba et al., 2012) under the Akaike Information Criterion (Posada and Buckley, 2004). The phylogenetic trees were constructed, respectively by maximum likelihood (ML) (Felsenstein, 1981) implemented in the software RaxmlGUI version 1.3 (Silvestro and Michalak, 2012) under default settings using the GTR+I model for 16S rRNA gene and the GTR+I+G model for single housekeeping gene and the concatenated genes (in the following order: *gyrB, rpoD, dnaK, trpB*, and *recA*). The type strain *Rhodovulum sulfidophilum* DSM 1374^T^ (Accession number: CP015418) was used as an outgroup in all phylogenetic analysis. Bootstrap confidence analysis was carried out with 1,000 replicates for evaluating the robustness of tree topologies. >70% Bootstrap values were shown at nodes of all trees. The bar represented nucleotide substitution rate (Knuc) units in each tree.

### Genome sequencing, dDDH and ANI calculation, and DNA G+C content

The representative strains were selected on the basis of MLSA analysis. The genome sequences of these strains were determined by Shanghai Majorbio Bio-pharm Technology Co., Ltd. using Solexa paired-end (500 bp library) sequencing technology. About 500 Mbp of clean data were generated with an Illumina/Solexa Genome Analyzer IIx (Illumina, SanDiego, CA), reaching approximately 100-fold coverage depth, for each strain. The high quality reads were assembled SOAPdenovo version 1.05 with default parameters. And then the assembled contigs were submitted in GenBank database, and the assigned accession numbers and assembly statistics of them were listed in Table S3. The dDDH and ANI values for representative strains were calculated using the genome-to-genome distance calculator website service (GGDC 2.1) with the recommended formula 2 (http://ggdc.dsmz.de/distcalc2.php) and the EzGenome web service (http://www.ezbiocloud.net/ezgenome/ani), respectively. A previous study demonstrated that the dDDH values at the upper 95% confidence interval (CI) were in better agreement with those of ANI for delineating species (Colston et al., 2014). Therefore, the upper 95% CI dDDH values were used for analysis in this study. Correlation analyses between the dDDH values and MLSA similarities were simulated using a nonlinear interpolation analysis method with the default option of the Curve Fitting Tool implemented in the commercially available software MATLAB version R2013a (MathWorks Inc., USA). Additionally, the genomic DNA G+C contents of representative strains were determined based on the respective genomic sequences.

### Morphological and physiological characterization

Cell and colony morphology, motility, Gram-staining, and anaerobic growth of representative strains were performed according to the previously described methods (Liu et al., 2017b). Catalase and oxidase activity was determined according to established procedures (Liu et al., 2016). The ranges and optimum of temperature, pH and NaCl were performed according to the methods previously described (Lai et al., 2014b). Other biochemical tests were carried out using API ZYM, API 20NE and API 20E strips (bioMérieux) according to the manufacturer's instructions, with the modification of adjusting the NaCl concentration to 3.0% in all tests.

### Fatty acid, polar lipid, and quinone characterization

For fatty acid analysis, the cells were harvested from the third quadrants on MA medium. The cells were saponified, methylated, and extracted using the standard MIDI (Sherlock Microbial Identification System, version 6.0B) protocol. The fatty acids were then analyzed by gas chromatography (Agilent Technologies 6850) (Sasser, 1990). Analysis of the respiratory quinones and polar lipids were carried out according to the previously described method (Collins, 1985; Kates, 1986).

## Results

### Individual gene analyses

Almost full-length 16S rRNA gene sequences and partial nucleotide sequences of five housekeeping genes, including *gyrB, rpoD, dnaK, trpB*, and *recA*, were determined for all strains. The features of each gene and five concatenated genes are displayed in Table 2.

**Table 2:** Characteristics of the 16S rRNA gene, single housekeeping gene and the concatenated genes from all strains.

**Locus**	**Length (bp)**	**No. of Alleles**	**Parsimony informative sites**		**Mean G+C content (%)**	**Similarities (%)**		***Ka*/*Ks***
			**No**.	**%**		**Range**	**Mean**	
16S rDNA	1430–1432^a^	10	48	3.36	54.9	95.9–100	99.0	–
*gyrB*	933–936^b^	21	197	21.1	62.9	83.1–100	92.9	0.072
*rpoD*	885	20	164	18.5	60.9	85.4–100	93.9	0.044
*dnaK*	918	21	142	15.5	60.6	85.0–100	94.9	0.036
*trpB*	927	20	196	21.1	64.6	84.6–100	93.3	0.043
*recA*	852	20	170	20.0	63.1	83.0–100	93.5	0.021
MLSA	4515–4518	22	869	19.2	62.4	84.8–100	93.7	–

a *Two base pairs (GC) insertion were found at the same position of 16S rRNA gene in strain MCCC 1A00513 and MCCC 1A03957, and thus the length of 16S rRNA gene sequence was 1,432 bp in the two strains*.

b *Three base pairs (GAG) insertion was observed at the same position of gyrB gene in strain MCCC 1A03957 and ES.031, and thus the length of gyrB gene sequence was 936 bp in two strains*.

**Figure 1:**
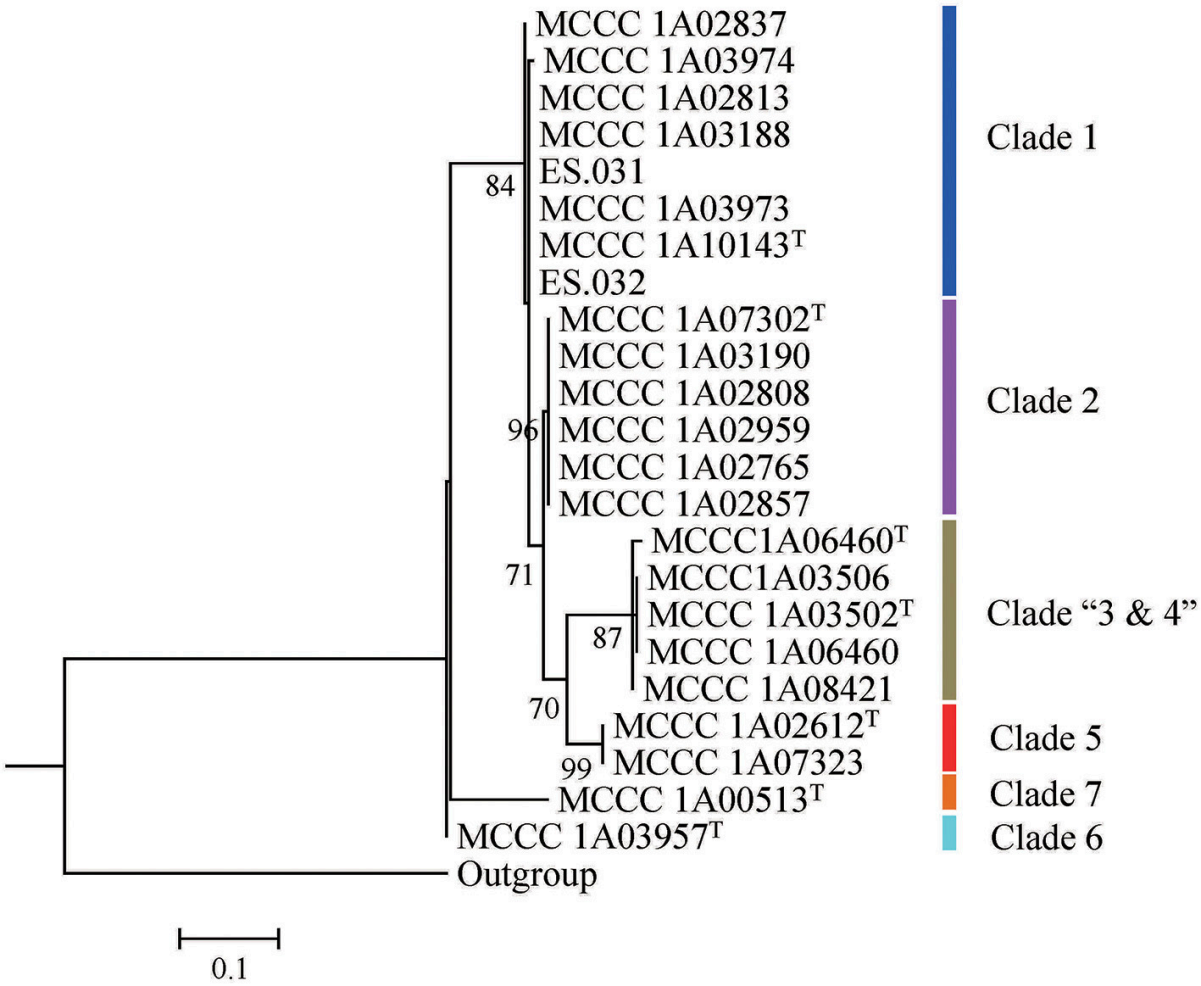
The ML tree based on 16S rRNA gene sequences.

The trimmed 16S rRNA gene sequences with 1,430/1,432 bp in length of all isolates were used for phylogenetic analysis. Among 23 strains, the number of alleles was ten, and the numbers and proportion of parsimony informative sites (S) were, respectively, 48 and 3.36%. The mean G+C content was 54.9 mol%. The similarities ranged from 95.9 to 100%, with a mean value of 99.0%. As shown in Figure 1, 23 strains in ML tree based on 16S rRNA gene can be grouped into six clades. The first clade, designated Clade 1, included two branches; the first branch only contained one strains; the second contained seven strains with two sequence types (ST) (type strain MCCC 1A10143^T^). The second clade, named Clade 2, comprised the type strain MCCC 1A07302^T^ and other five strains, exhibiting the same ST. The third clade, called Clade “3 and 4,” consisted of the two type strains (MCCC 1A06460^T^ and MCCC 1A03502^T^) and other three strains, with three STs. The remaining three clades, marked Clade 5, 6, and 7, corresponded to the three species *T. atlantica, T. dalianensis*, and *T. indica*. The pairwise similarities of 16S rRNA gene sequences among all strains used in this study were higher than the threshold value of 97% that is used as the standard of the species boundary (Stackebrandt and Goebel, 1994), with exception of the type strain *T. indica* MCCC 1A00513^T^ and 11 strains belonging to Clade 2, 3, and 4 (referred to MLSA analysis below). Therefore, 16S rRNA gene is inadequate to discriminate closely related species and strains.

The sequence lengths for the five housekeeping genes (Table 2) were from 852 bp (*recA*) to 933/936 bp (*gyrB*). Among 23 strains, the numbers of alleles (20–22) for these genes were roughly the same. The *gyrB* gene possessed the most parsimony informative sites with 197, followed by *trpB* with 196, *recA* with 170, *rpoD* with 164, and *dnaK* with 142. The percentages of parsimony informative sites for all genes were higher than 15%, suggesting more genetic information against 16S rRNA gene. The mean G+C contents of all genes were from 60.6 to 64.6 mol%. The ranges and mean values of the five gene similarities were not remarkable difference. In addition, a slight variation of the *Ka*/*Ks* ratio for the five housekeeping genes was observed in Table 2, suggesting that two genes, *dnaK* and *recA*, were under a higher negative selective pressure relative to the three genes, *rpoD, trpB*, and *gyrB*. However, all values were far below 1, indicating that these genes were under strong purifying selection (Hurst, 2002) and thereby suitable for MLSA. These results clearly demonstrated that each housekeeping gene owns a much higher discriminatory power compared to the 16S rRNA gene.

Test for substitution saturation of all genes sequences showed that they were not saturated in substitutions. Meanwhile, no recombination events were found in any of them, as determined by RDP 4.16. These results demonstrated that all gene sequences could provide essential phylogenetic information.

In comparison with 16S rRNA gene tree, all single-gene trees based on nucleotide sequences showed much higher resolution on the basis of more branches and higher bootstrap values (Figure S1). Moreover, these single-gene trees are generally of the same topology with seven clades (see the detailed description in MLSA section). However, some differences were observed in the deep branches of trees. For example, Clade 6 was a sister taxon to Clade 7 in the trees based on *gyrB, rpoD, dnaK*, or *recA* gene, while it was a deep branch in the tree based on *trpB* gene, and far related to Clade 7 (Figure S1). Clade 5 exhibited the varied positions in the five trees (Figure S1). In addition, some bacteria in Clade 1 and Clade 2 shifted from one position to another in different single-gene trees. Such as in the tree of *gyrB*, strain MCCC 1A03974 neighbored with MCCC 1A10143^T^, while it is by side of MCCC 1A02813 in the *recA* tree. Therefore, the single housekeeping gene cannot accurately infer the phylogenetic relationships of some members of the genus *Thioclava*, although it is effective in differentiating different species, and even different strains within a species.

### MLSA

The concatenated sequences of the five housekeeping genes with 4,518 bp in length contained 22 alleles and 869 parsimony informative sites (19.2%) with a mean G+C content of 62.4 mol% (Table 2). The MLSA similarities among all tested strains ranged from 84.8 to 100%, with a mean value of 93.7% (Table S4). The MLSA tree showed a similar topology to the single housekeeping gene tree as described above, but showed much higher bootstrap values.

**Figure 2:**
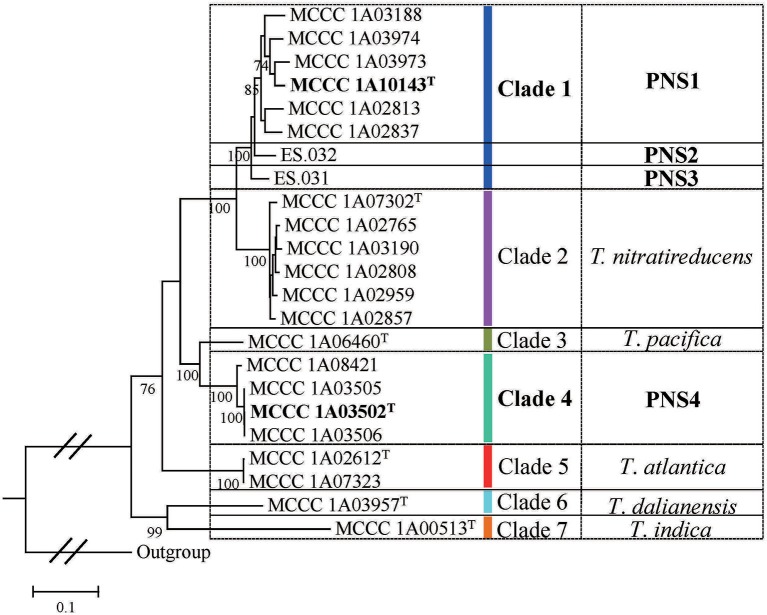
The ML tree based on the five concatenated housekeeping genes sequences.

The 23 strains were divided into seven clades denoted as Clade 1 to Clade 7 in the MLSA tree (Figure 2). Specifically, Clade 1 had eight branches with eight STs, each of which represents a single strain. The Clade 2 was separated into six branches with six STs corresponding to the type strain MCCC 1A07302^T^ and other five strains. The Clade 3 only contained the type strain MCCC 1A06460^T^. The Clade 4 was split into three branches including four strains with three STs. The Clade 5 included two strains with the same genotype, the type strain MCCC 1A02612^T^ and MCCC 1A07323. The Clade 6 and Clade 7 were, respectively represented by the type strains MCCC 1A03957^T^ and MCCC 1A00513^T^. Of them, bacteria in Clade 1 and Clade 4 cannot be allocated to any previously described species, and therefore, they are potential novel species.

### General genome features

The general genome features of the eight newly sequenced strains and seven previously sequenced strains are summarized in Table S3. The genome sizes of these strains ranged from 3,653,331 to 4,962,820 bp. Contigs number ranged from one to 105, depending on the strain. The longest contigs of the genome was 3,938,466 bp from strain ES.032. The N50 length for these contigs showed significant differences, ranging from 116,979 to 2,098,934. The G+C content of these strains ranged from 60.3 mol% (MCCC 1A00513^T^) to 65.3 mol% (MCCC 1A02612^T^).

### The dDDH and ANI values and comparison with MLSA

In order to further confirm the results of MLSA, the genome sequences of representative strains from seven clades were determined (at least one per clade). The pairwise dDDH and ANI values of 15 genome sequences were, respectively, calculated according to the method mentioned above, including five from GenBank database and two from IMG database. As listed in Table S5, most of dDDH values were <70%, that is often used as the gold standard for bacterial species definition (Wayne et al., 1987). Likewise, almost all ANI values were under a narrowed boundary of 95–96% (Table S6), which was proposed as the species definition for prokaryotes (Richter and Rossello-Mora, 2009). It is noteworthy that the dDDH values among the six MCCC strains in Clade 1 were slightly higher than 70%. Furthermore, the ANI values among the six strains were above 96%. Therefore, they belonged to the same species. Other two strains in Clade 1, strain ES.031 and ES.031, each represented a putative novel species on the basis of the dDDH and ANI values. Evidently, Clade 1 comprised three putative novel species. For other six clades, each represented one unique species, among which Clade 4 represented a novel species. Therefore, a total of four putative novel species were recommended in this study. Here, only two novel isolates from our group, one in Clade 1, and the one in Clade 4, were characterized further in taxonomy, and named as *Thioclava sediminum* sp. nov. and *Thioclava marinus* sp. nov. as below. The two novel species represented by ES.031 and ES.032 in Clade 1 had no culture in our lab and thus could not be characterized in the present study.

**Figure 3:**
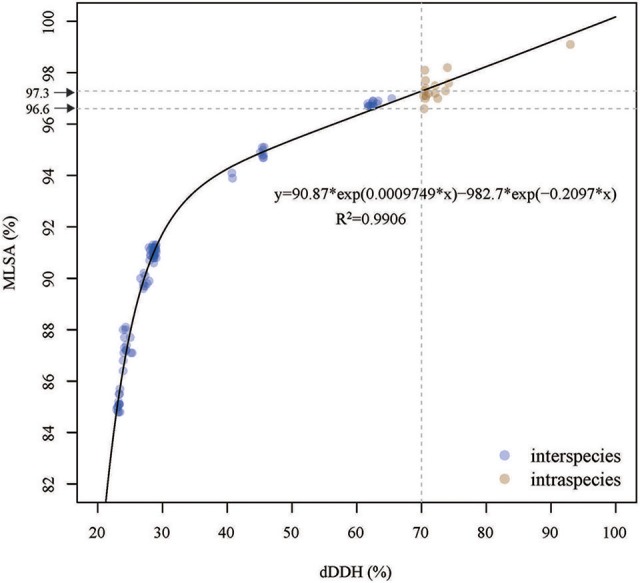
The Correlation analysis between the dDDH values and MLST similarities for resprestative *Thicolava* strains. The vertical line indicates the 70% dDDH threshold. The horizontal line (y = 97.3) indicates the estimated MLSA similarity threshold corresponding to 70% dDDH threshold.

The correlation between the dDDH values and MLSA similarities for 15 strains was determined by a nonlinear interpolation analysis method. The dDDH values were highly correlated with the MLSA similarities (*R*^2^ = 0.9906). Based on the simulative equation of y = 90.87^*^exp(0.0009749^*^x)−982.7^*^exp(−0.2097^*^x), a 70% dDDH value was equivalent to a 97.3% MLSA similarity (Figure 3), which could be used to be species threshold for the members of the genus *Thioclava*. However, a gap of MLSA similarities from 97.3 to 96.6% was shown in Figure 3. Concretely speaking, there were three situations based on the current analyses in this study: (1) the strains were definitely the same species when MLSA similarities were above 97.3%; (2) the strains were the same or different species when they range from 97.3 to 96.6%; (3) the strains were affirmably different species when they were below 96.6%. As a result, the MLSA cutoff should be used as a soft rather than hard boundry for demarcating species within the genus *Thioclava*.

### Phenotypic characteristics of *Thioclava sediminum* and *Thioclava marinus*

The cell shape of *Thioclava sediminum* TAW-CT134^T^ and *Thioclava marinus* 11.10-0-13^T^, are the same as those of reference strains (short rods) (Figure S2). By contrast, the type strain *T. pacifica* MCCC 1A06460^T^ varied from long filaments with swollen ends, clustered in aggregates, to single small rods (Sorokin et al., 2005). Colonies of all strains are circular, non-translucent, and smooth with entire margins after 3 days at 28°C. *T. sediminum* TAW-CT134^T^ is motile by means of a polar flagellum, while *T. marinus* 11.10-0-13^T^ does not possess flagella. All strains are Gram-staining-negative, aerobic, catalase- and oxidase-positive. The growth and optimal temperature, pH and NaCl ranges for growth of these two strains are similar to the ones of reference strains (Liu et al., 2017b). In the API ZYM, API 20NE and API 20E tests, all strains present similar physiological characteristics, indicating that the two strains belong to the genus *Thioclava*. However, as shown in Table 3 and in the species description below, some morphological and physiological characteristics distinguish both strains from other known species.

**Table 3:** Characteristics that differentiate the two novel strains from the reference strains.

**Characteristics**	**1**	**2**	**3**	**4**	**5**	**6**	**7**
Motile	+	−	+	+	+	−	−
Cell length (μm)	1.0–1.2	1.4–1.6	1.1–1.2	1.2–2.0	Nd	1.1–1.2	1–1.2
Cell width (μm)	0.6–0.7	0.5–0.6	0.6–0.7	0.7–0.8	Nd	0.7–0.8	0.7–0.8
Temperature range (°C)	10–43	10–45	10–43	4–41	15–47	4–37	10–41
Optimum temperature	28–32	28–32	28	28–37	35	28	28–32
NaCl range (%, w/v)	0–12	0–12	0–12	0.5–12	1–8	0.5–15	0–18
Optimum NaCl	3	1–3	3–4	3–5	3–4	3	3
**API ZYM**							
Lipase (C14)	−	+	+	+	−	+	+
α-Galactosidase	+	−	+	−	−	+	+
β-Galactosidase	+	−	+	−	−	+	+
β-Glucosidase	+	+	+	+	+	+	−
**API 20NE**							
Reduction of nitrate	+	+	+	+	−	+	+
Denitrification	−	+	−	−	−	−	+
D-Glucose fermentation	−	−	+	+	−	−	−
Urease	+	+	+	+	+	+	−
β-Glucosidase	+	−	+	+	+	+	−
β-Galactosidase	+	−	+	−	−	+	+
Utilization of L-arabinose	+	−	+	+	−	+	−
D-mannose	+	+	+	+	−	+	+
Potassium gluconate	+	+	+	+	−	+	+
Malic acid	+	+	+	+	+	+	−
Trisodium citrate	+	−	+	+	+	+	+
**API 20E**							
β-Galactosidase	+	−	+	−	−	+	+
Urease	+	+	+	+	+	+	−
Acid production of glucose	−	−	+	+	−	−	−
saccharose	−	−	−	−	+	−	−
**G+C content (mol%)**	64.0	64.1	63.8	65.3	63.9	62.5	60.3

*Strains: 1, TAW-CT134^T^; 2, 11.10-0-13^T^; 3, MCCC 1A07302^T^; 4, MCCC 1A02612^T^; 5, MCCC 1A06460^T^; 6, MCCC 1A03957^T^; 7, MCCC 1A00513^T^. The data of cell length and width, the ranges and optimum of temperature and NaCl for the latter five strains were from the original identification results unless otherwise stated. The data of API ZYM, API 20NE, and API 20E tests for the latter five strains were obtained from the published the results by us. Characteristics are scored as: +, positive; −, negative; v, variable; Nd, no data*.

Below are the chemotaxonomic features of the two strains, analyzed in parallel with other reference strains. As a result, the composition of fatty acids for the two novel strains are almost consistent with those of closely related type strains, with Summed Feature 8 (C_18:1_ω6*c* and/or C_18:1_ω7*c*) as the major component (Table S7), and supporting their allocation in the genus *Thioclava* (Liu et al., 2017b). However, the proportions of fatty acids varied to some extent among them (Table S7). The quinone of the two novel strains is identified as Q-10, which is in accordance with data from reference strains (Liu et al., 2017b). The types of polar lipids for the two novel species are almost identical, with the presence of phosphatidylglycerol (PG), phosphatidylethanolamine (PE), unidentified glycolipid (GL), and several unidentified phospholipids (PLs) (Figure S3). The distinctive feature is the presence of aminophospholipid in type strain MCCC 1A02612^T^ (Lai et al., 2014a) and MCCC 1A03957^T^ (Zhang et al., 2013). The DNA G+C content of two novel strains TAW-CT134^T^ and 11.10-0-13^T^ are, respectively, 64.0 and 64.1 mol%, which are in the same range than the ones of the five type strains (60.3–65.3 mol%) (Liu et al., 2017b).

## Discussion

The members of the genus *Thioclava* are ubiquitous in marine environments and distributed in a variety of ecological niches, such as surface and deep seawater, deep sea sediment, halobios in global oceans like the Pacific Ocean, the Atlantic Ocean, the Indian Ocean and even the Arctic Ocean (Wang et al., 2012, 2014; Rowe et al., 2015). However, the phylogeny of bacteria within this genus has been not well understood so far. In this report, a MLSA scheme based on five housekeeping genes with *gyrB, rpoD, recA, trpB*, and *dnaK* was established for referring the phylogeny of the species known and other putative novel species.

As mentioned above, 16S rRNA gene as a phylogenetic marker often provides low resolution in differentiation of some strains and species. Mutiple previous studies demonstrated that some species shared >97%, and even 100% of 16S rRNA gene sequences (Das et al., 2014; Yarza et al., 2014; Liu et al., 2015b). Likewise, in this study, we found that the strains affiliated to three species in Clade 1 sharing 99.9% similarities between 16S rRNA gene sequences, which is quite higher than the traditionally recognized threshold of 97% (Stackebrandt and Goebel, 1994), even the latest recommended threshold of 98.65% (Kim et al., 2014). Therefore, we should keep in mind the fact that 16S rRNA gene has been widely used to identify phylogenetic relationships for all microorganisms on earth, but is not good indicator in determining the accurate taxonomic position of some bacteria, for example the members of the genus *Thioclava* in this study.

In recent years, the MLSA approach has shed new light on prokaryotic systematics and phylogeny (Martens et al., 2008; Vinuesa, 2010; Glaeser and Kämpfer, 2015), and has been extensively used in the classification and identification of diverse bacteria. For example, the phylogenetic relationships of the type strains for 107 species within the genus *Pseudomonas* was performed using an atypia MLSA based on the four core housekeeping genes (16S rRNA gene, *gyrB, rpoB*, and *rpoD*) and on this basis a 97% MLSA similarity was proposed to be as a threshold for species delineation (Mulet et al., 2010). During the process of investigation of the evolution and taxonomy of native mesorhizobia nodulating medicinal *Glycyrrhiza* species in China, the eight tested strains actually belonged to the same putative new species of the genus *Mesorhizobium* by MLSA (Ampomah et al., 2017). It was stated that the MLSA scheme can be applied in diverse *Streptomyces* clades that contain species with high 16S rRNA gene sequence similarities; a 0.007 of nucleotide sequence distance of the five genes corresponding to a 70% DDH value could be considered as the species cut-off for the whole genus (Rong and Huang, 2010, 2012). Similarly, a well-established MLSA scheme for the genus *Thioclava* in our study proved a powerful method for discrimination of the *Thioclava* bacteria. Moreover, a 97.3% MLSA similarity was proposed as the soft species threshold of the genus *Thioclava* based on the correlation analysis between dDDH and MLSA, leading to the discovery of four putative novel species.

Although MLSA is an easy, practical, reliable, and robust technique for identification of bacterial isolates as alternatives to 16S rRNA gene sequence (Glaeser and Kämpfer, 2015), it lacks a uniform cut-off value for identifying them to the species level (Vinuesa, 2010; Glaeser and Kämpfer, 2015). Over the years, many different threshold values of MLSA for species distinction have been recommended in previous studies, for example, 96.16–97.32% for the genus *Thalassospira* (Lai et al., 2014c), 97.74% for the *Bacillus cereus* group (Liu et al., 2015b), 97% for the *Burkholderia cepacia* complex (Peeters et al., 2013) and in the genus *Pseudomonas* (Mulet et al., 2010), and 97.3% for the genus *Thioclava* in the present study. Moreover, different housekeeping genes from different strains and with analytical methodologies make it difficult to set up a canonical scheme. In addition, MLSA is limited by a few reference sequences of housekeeping genes against a tremendous amount of commonly used rRNA gene sequences in public database. Therefore, MLSA still needs improvements in order to make the application more feasible and generally applicable in the future. Luckily, the advent of the genome sequencing era and the appearance of dDDH and ANI will contribute to compensate the shortcomings of MLSA (Konstantinidis and Tiedje, 2005; Liu et al., 2015b; Hahnke et al., 2016).

In summary, the bacterial phylogeny and taxonomy of the genus *Thioclava* were determined by a newly established MLSA in combination with dDDH, ANI, and other approaches in this report. The four novel species have been added to the genus with five other established species. We established a robust framework for the discrimination of *Thioclava* isolates, which will provide a great avenue for a better understanding their geographic distribution and ecological roles in diverse marine environments.

### Description of *Thioclava sediminum* sp. nov

*Thioclava sediminum* (se.di.mi′num. L. gen. pl. n. sediminum of sediments, pertaining to source of isolation).

Cells are Gram-staining-negative, aerobic, short-rod-shaped, motile by means of a polar flagellum, 0.6–0.7 μm in width and 1.0–1.2 μm in length (Figure S2). Colonies are light brown-colored, circular, non-translucent, smooth with entire margins, and approximately 1–2 mm in diameter on MA medium after 3 days at 28°C. Growth occurs at 10–43°C (optimum 28–32°C), at pH 6–10 (optimum, pH 7-8), and in 0–12% NaCl (optimum 3%, w/v). Catalase and oxidase are positive. In the API ZYM tests, positive for alkaline phosphatase, esterase (C4), esterase lipase (C8), leucine arylamidase, valine arylamidase, naphthol-AS-BI-phosphohydrolase, acid phosphatase, α-galactosidase, β-galactosidase, α-glucosidase, and β-glucosidase; negative for lipase (C14), cystine arylamidase, trypsin, α-chymotrypsin, β-glucuronidase, N-acetyl-β-Glucosaminidase, α-mannosidase, and α-fucosidase. In the API 20NE tests, positive for nitrate reduction, urease, β-glucosidase (Aesculin hydrolysis), β-galactosidase, utilization of D-glucose, L-arabinose, D-mannose, D-mannitol, D-maltose, potassium gluconate, malic acid, and trisodium citrate; negative for denitrification, indole production, D-glucose fermentation, arginine dihydrolase, gelatin hydrolysis, utilization of N-acetyl-glucosamine, capric acid, adipic acid, and phenylacetic acid. In the API 20E tests, positive for β-galactosidase, citrate utilization and urease; negative for arginine dihydrolase, lysine decarboxylase, ornithine decarboxylase, H_2_S production, tryptophane deaminase, indole production, acetoin production (Voges Proskauer), gelatinase, acid production of glucose, mannitol, inositol, sorbitol, rhamnose, saccharose, melibiose, amygdalin and arabinose. The major fatty acid is Summed Feature 8. The isoprenoid quinone is Q-10. The polar lipids comprised PE, PG, GL, and PLs.

The type strain, TAW-CT134^T^ (=MCCC 1A10143^T^ = LMG 29615^T^), was isolated the coastal sediment around Xiamen Island, Fujian province, China. The DNA G+C content of the type strain is 64.0 mol%.

### Description of *Thioclava marinus* sp. nov

*Thioclava marinus* (ma.ri′nus. L. masc. adj. marinus of the sea, the origin of the sample from which the type strain was isolated).

Cells are Gram-staining-negative, aerobic, short-rod-shaped, non-motile, 0.5–0.6 μm in width and 1.4–1.6 μm in length (Figure S2). Colonies are light yellow-colored, circular, non-translucent, smooth with entire margins and approximately 1–2 mm in diameter on MA medium after 3 days at 28°C. Growth occurs at 10–45°C (optimum 28–32°C), at pH 6-9 (optimum, pH 7-8), and in 0–12% NaCl (optimum 1–3%, w/v). Catalase and oxidase are positive. In the API ZYM tests, positive for alkaline phosphatase, esterase (C4), esterase lipase (C8), lipase (C14), leucine arylamidase, valine arylamidase, acid phosphatase, naphthol-AS-BI-phosphohydrolase, α-glucosidase, and β-glucosidase; negative for cystine arylamidase, trypsin, α-chymotrypsin, α-galactosidase, β-galactosidase, N-acetyl-β-Glucosaminidase, β-glucuronidase, α-mannosidase, and α-fucosidase. In the API 20NE tests, positive for nitrate reduction, denitrification, urease, utilization of D-glucose, D-mannose, D-mannitol, D-maltose, potassium gluconate, and malic acid; negative for indole production, D-glucose fermentation, arginine dihydrolase, β-glucosidase (Aesculin hydrolysis), gelatin hydrolysis, β-galactosidase, utilization of L-arabinose, N-acetyl-glucosamine, capric acid, adipic acid, trisodium citrate, and phenylacetic acid. In the API 20E tests, positive for citrate utilization and urease; negative for β-galactosidase, arginine dihydrolase, lysine decarboxylase, ornithine decarboxylase, H_2_S production, tryptophane deaminase, indole production, acetoin production (Voges Proskauer), gelatinase, acid production of glucose, mannitol, inositol, sorbitol, rhamnose, saccharose, melibiose, amygdalin, and arabinose. The major fatty acid is Summed Feature 8. The isoprenoid quinone is Q-10. The polar lipids comprised PE, PG, GL, and PLs.

The type strain, 11.10-0-13^T^ (=MCCC 1A03502^T^ = LMG 29618^T^), was isolated from the coastal seawaters of Yellow Sea, China. The DNA G+C content of the type strain is 64.1 mol%.

## Author contributions

YL and ZS conceived the experiment. YL conducted the experiment, analyzed the result and wrote the paper. YL, QL, and ZS reviewed the manuscript.

### Conflict of interest statement

The authors declare that the research was conducted in the absence of any commercial or financial relationships that could be construed as a potential conflict of interest.
