# Supplementary material for: A Multilocus Sequence Analysis Scheme for Phylogeny of Thioclava Bacteria and Proposal of Two Novel Species
Source: Front Microbiol. 2017 Jul 13;8:1321. doi: 10.3389/fmicb.2017.01321 (PMC5508018; doi:10.3389/fmicb.2017.01321)
Supplement: Supplementary file 1 [file DataSheet1.DOCX]

**A Multilocus Sequence Analysis Scheme for Phylogeny of *Thioclava* Bacteria and Proposal of Two Novel Species**

Yang Liu^1, 2^, Qiliang Lai^2^, Zongze Shao^1, 2,^*

^1^ School of Municipal and Environmental Engineering, Harbin Institute of Technology, Harbin 150090, China.

^2^ State Key Laboratory Breeding Base of Marine Genetic Resources; Key Laboratory of Marine Genetic Resources, Third Institute of Oceanography, SOA; Collaborative Innovation Center for Exploitation and Utilization of Marine Biological Resources; Key Laboratory of Marine Genetic Resources of Fujian Province, Xiamen 361005, China.

*Corresponding author: Zongze Shao, E-mail: shaozz@163.com.

Types of paper: Original Research

Section: Evolutionary and Genomic Microbiology

Running Title: Phylogeny of *Thioclava* Bacteria

**Supplementary Data**

**
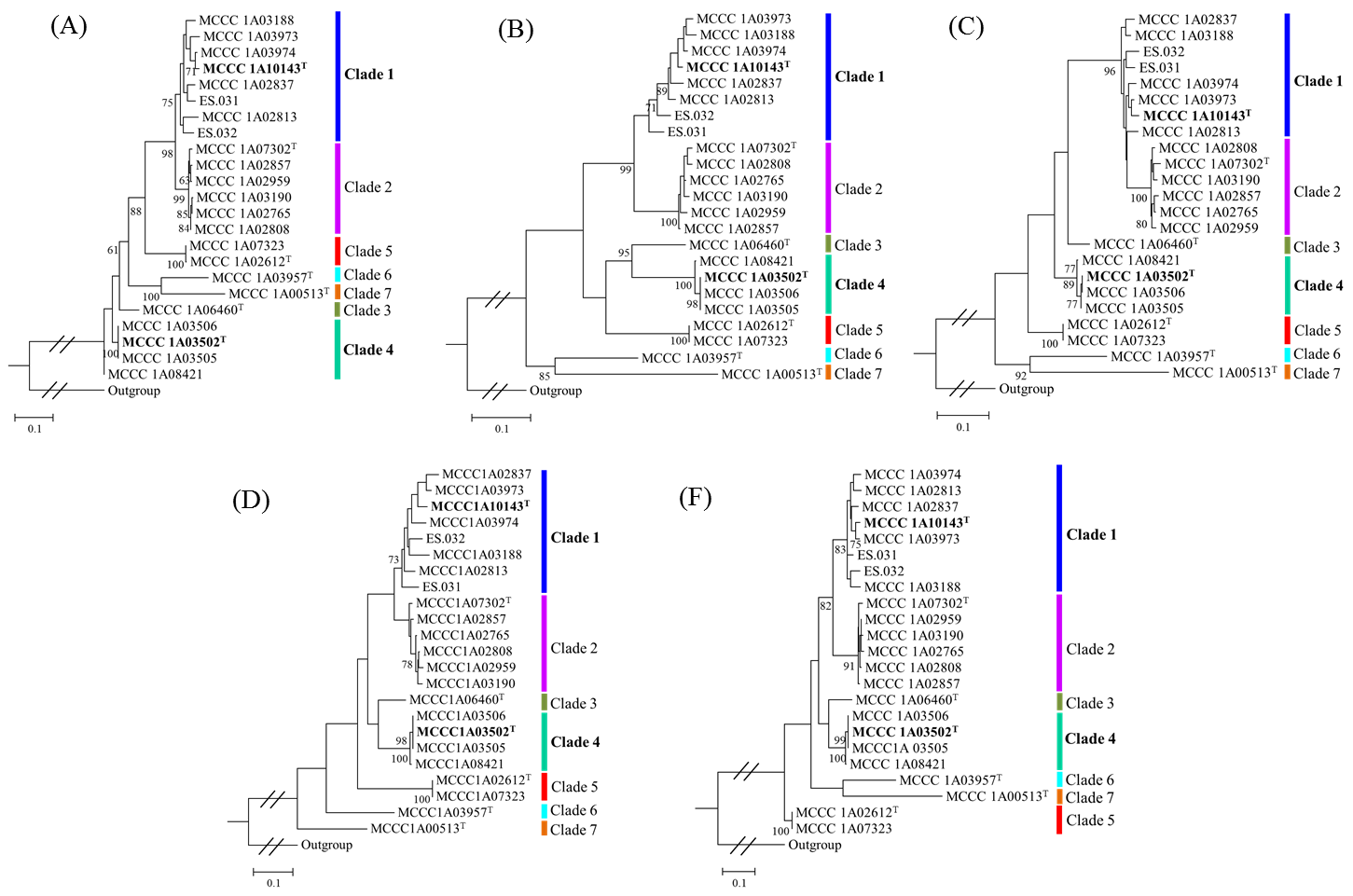
**

**Figure S1** The ML trees based on *gyrB* (A), *rpoD* (B), *dnaK* (C), *trpB* (D), and *recA* (E) gene sequences

**
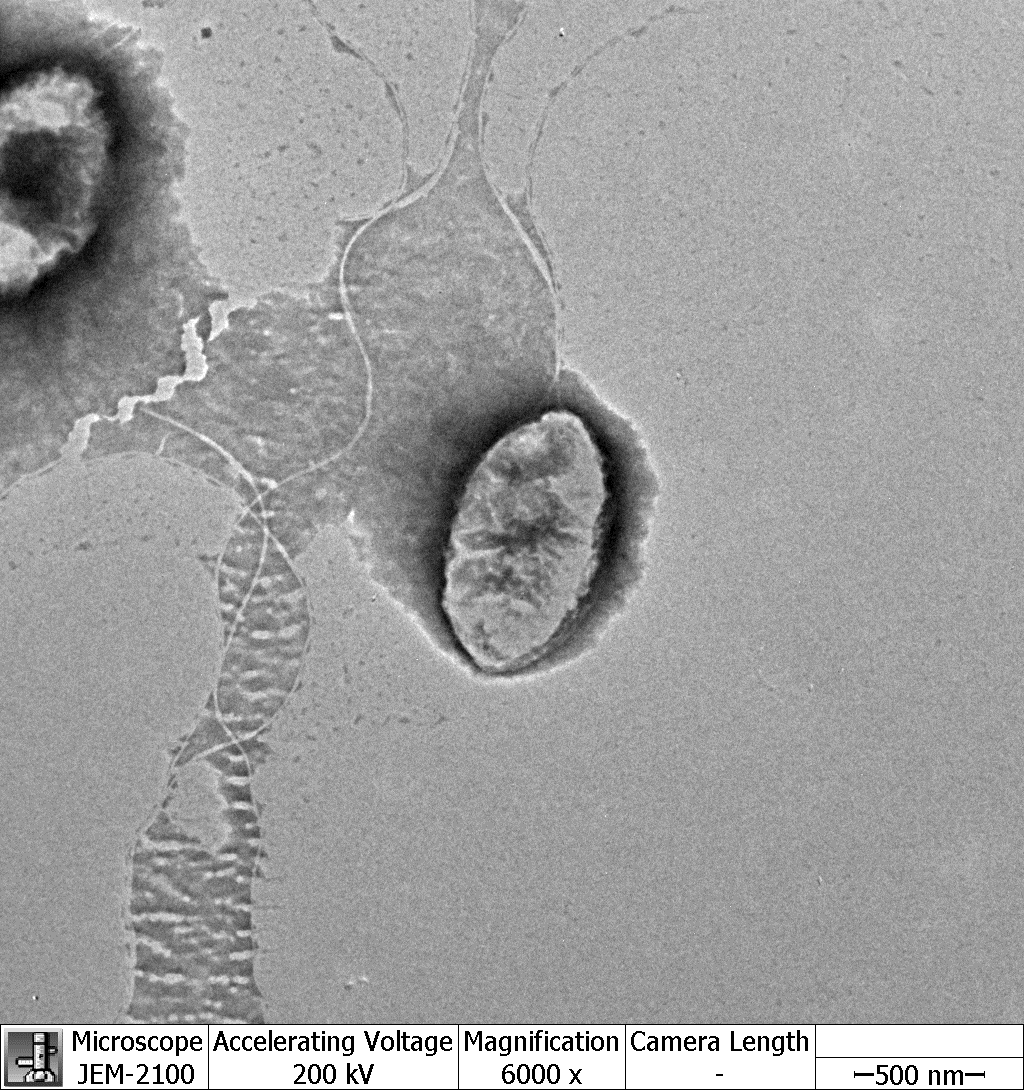
** **
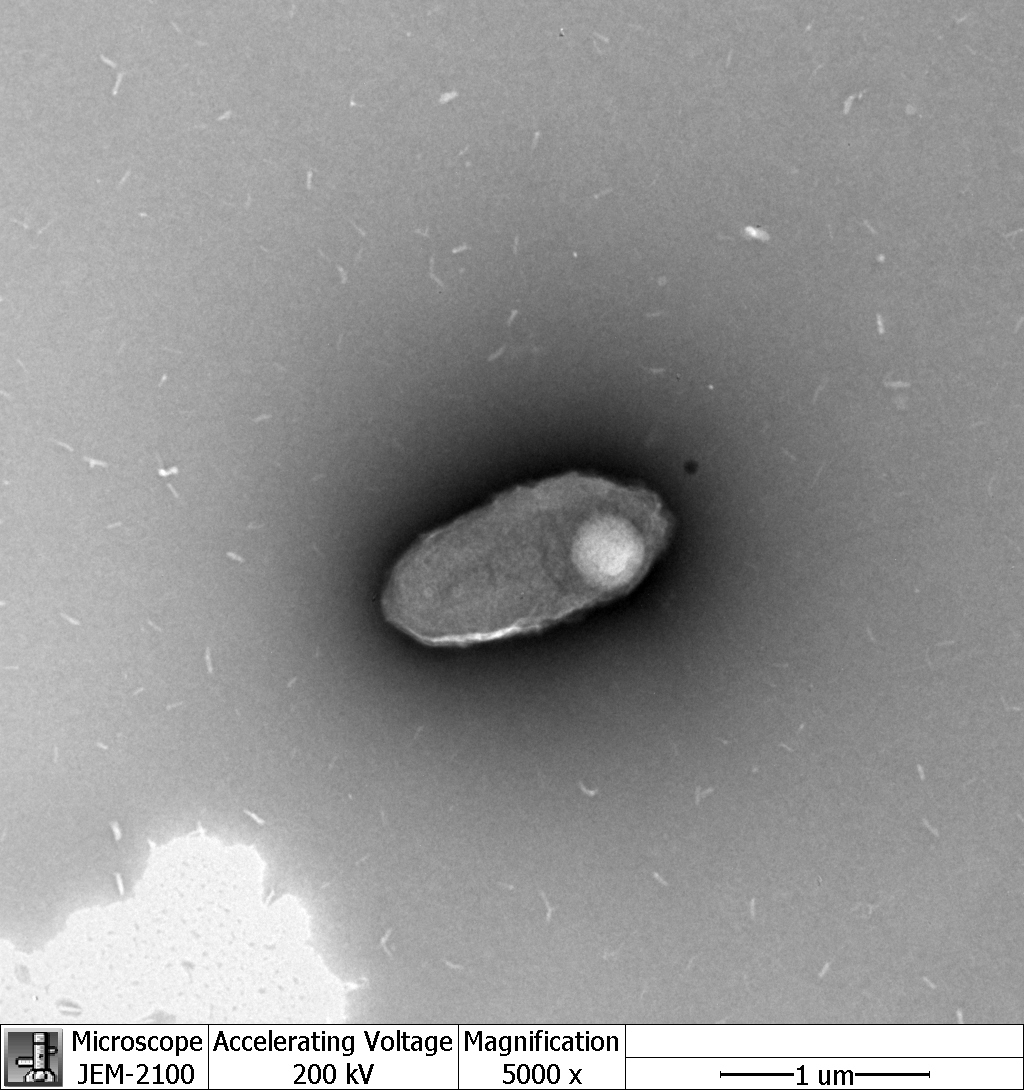
**

**B**

**A**

**Figure S2.** Transmission electron micrographs of type strain TAW-CT134^T^ (A) and 11.10-0-13^T^ (B) on MA at 28°C for 24 h.

**
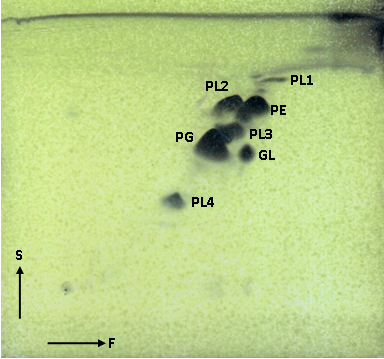

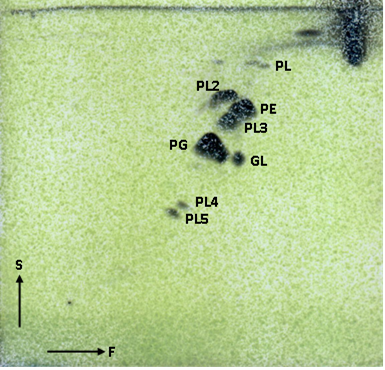
**

**A**

**B**

**Figure** **S3** Polar lipids profile of type strain TAW-CT134^T^ (A) and 11.10-0-13^T^ (B).

Abbreviations: PE, phosphatidylethanolamine; PG, phosphatidylglycerol; GL, glycolipid; PLs, unidentified phospholipids; F, first dimension of TLC; S, second dimension of TLC.

**Table S1** Characteristics of the primers in this study

| Gene | Primer name | Sequence (5' to 3') | Amplified fragment length (bp) | Annealing temperature (^o^C) | Extension time (s) |
| --- | --- | --- | --- | --- | --- |
| 16S rDNA | 27F^†^ | AGAGTTTGATCCTGGCTCAG | 1432 | 55 | 90 |
|  | 1492R^†^ | ACGGCTACCTTGTTACGACT |  |  |  |
|  | P300^‡^ | CCAGACTCCTACGGGAGGCAGC |  |  |  |
|  | RP500^‡^ | CGTATTACCGCGGCTGCTGGCA |  |  |  |
|  | P800^‡^ | TGCATGGCTGTCGTCAGCTCGTG |  |  |  |
| *gyrB* | UP-1^†^ | GAAGTCATCATGACCGTTCTGCAYGCNGGNGGNAARTTYGA | 1200 | 58 | 70 |
|  | UP-2R^†^ | AGCAGGGTACGGATGTGCGAGCCRTCNACRTCNGCRTCNGTCAT |  |  |  |
|  | UP-1S^‡^ | GAAGTCATCATGACCGTTCTGCA |  |  |  |
| *rpoD* | Thio-rpoD-1019F^†‡^ | CCGCAAGATCATGTCGAT | 970 | 56 | 60 |
|  | Thio-rpoD-1988R^†^ | TGCTTGAGCTTGCGCAG |  |  |  |
| *dnaK* | Thio-dnaK-420F^†‡^ | CGCATCATCAACGAGCC | 998 | 46 | 60 |
|  | Thio-dnaK-1417R^†^ | GCCTGGATCGTGATCTT |  |  |  |
| *trpB* | Thio-trpB-90F^†‡^ | CTGATGCCGCTGATCCT | 1073 | 46 | 60 |
|  | Thio-trpB-1162R^†^ | TTCATCACGATGATATG |  |  |  |
| *recA* | Thio-recA-38F^†^ | CAAGGCAGACAAGCAAAAGGCG | 963 | 50 | 60 |
|  | Thio-recA-1000R^†‡^ | TTGTCCTCGATCTCATAGGC |  |  |  |

^†^: Amplification primer. ^‡^: Sequencing primer.

Arrreviation to represent ambiguity in sequence of *gyrB* gene primer: Y=C or T, R=A or G, N=A or T or C or G.

**Table S2** The accession numbers of the gene sequences for 23 strains

| MCCC No. | 16S rDNA | *gyrB* | *rpoD* | *dnaK* | *trpB* | *recA* |
| --- | --- | --- | --- | --- | --- | --- |
| MCCC 1A00513^T^ | KX618918 | KX618964 | KX619010 | KX618941 | KX619033 | KX618987 |
| MCCC 1A02612^T^ | KX618919 | KX618965 | KX619011 | KX618942 | KX619034 | KX618988 |
| MCCC 1A02765 | KX618920 | KX618966 | KX619012 | KX618943 | KX619035 | KX618989 |
| MCCC 1A02808 | KX618921 | KX618967 | KX619013 | KX618944 | KX619036 | KX618990 |
| MCCC 1A02813 | KX618922 | KX618968 | KX619014 | KX618945 | KX619037 | KX618991 |
| MCCC 1A02837 | KX618923 | KX618969 | KX619015 | KX618946 | KX619038 | KX618992 |
| MCCC 1A02857 | KX618924 | KX618970 | KX619016 | KX618947 | KX619039 | KX618993 |
| MCCC 1A02959 | KX618925 | KX618971 | KX619017 | KX618948 | KX619040 | KX618994 |
| MCCC 1A03188 | KX618926 | KX618972 | KX619018 | KX618949 | KX619041 | KX618995 |
| MCCC 1A03190 | KX618927 | KX618973 | KX619019 | KX618950 | KX619042 | KX618996 |
| MCCC 1A03502^T^ | KX618928 | KX618974 | KX619020 | KX618951 | KX619043 | KX618997 |
| MCCC 1A03505 | KX618929 | KX618975 | KX619021 | KX618952 | KX619044 | KX618998 |
| MCCC 1A03506 | KX618930 | KX618976 | KX619022 | KX618953 | KX619045 | KX618999 |
| MCCC 1A03957^T^ | KX618931 | KX618977 | KX619023 | KX618954 | KX619046 | KX619000 |
| MCCC 1A03973 | KX618932 | KX618978 | KX619024 | KX618955 | KX619047 | KX619001 |
| MCCC 1A03974 | KX618933 | KX618979 | KX619025 | KX618956 | KX619048 | KX619002 |
| MCCC 1A06460^T^ | KX618934 | KX618980 | KX619026 | KX618957 | KX619049 | KX619003 |
| MCCC 1A07302 | KX618935 | KX618981 | KX619027 | KX618958 | KX619050 | KX619004 |
| MCCC 1A07323^T^ | KX618936 | KX618982 | KX619028 | KX618959 | KX619051 | KX619005 |
| MCCC 1A08421 | KX618937 | KX618983 | KX619029 | KX618960 | KX619052 | KX619006 |
| MCCC 1A10143^T^ | KX618938 | KX618984 | KX619030 | KX618961 | KX619053 | KX619007 |
| ES.032 | KX618939 | KX618985 | KX619031 | KX618962 | KX619054 | KX619008 |
| ES.031 | KX618940 | KX618986 | KX619032 | KX618963 | KX619055 | KX619009 |

**Table S3** The accession numbers of the genome sequences for 15 representative strains

| MCCC No./Names | Accession numbers | Size (bp) | Contig number | Longest contig | N50 length | G+C mol% |  |
| --- | --- | --- | --- | --- | --- | --- | --- |
| MCCC 1A03973 | MPZT00000000^﹟^ | 4,361,606 | 14 | 1,126,644 | 649,591 | 63.8 |  |
| MCCC 1A10143^T^ | MPZV00000000^﹟^ | 4,131,279 | 8 | 1,602,917 | 1,030,685 | 64.0 |  |
| MCCC 1A03974 | MPZU00000000^﹟^ | 4,330,998 | 14 | 1,538,565 | 697,658 | 63.9 |  |
| MCCC 1A03188 | MPZR00000000^﹟^ | 4,301,526 | 34 | 640,128 | 444,676 | 63.9 |  |
| MCCC 1A02813 | MPZQ00000000^﹟^ | 4,580,168 | 70 | 1,040,122 | 354,167 | 63.6 |  |
| MCCC 1A02837 | MPZX00000000^﹟^ | 4,060,064 | 5 | 1,533,108 | 1,458,483 | 64.1 |  |
| ES.031 | 2615840533^§^ | 4,019,144 | 1 | - | - | 64.2 |  |
| ES.032 | 2615840522^§^ | 4,962,820 | 2 | 3,938,466 | 1,024,354 | 62.8 |  |
| MCCC 1A07302^T^ | MQMR00000000^﹩^ | 4,185,626 | 59 | 608,220 | 279,862 | 63.8 |  |
| MCCC 1A06460^T^ | AUND00000000^﹩^ | 3,728,293 | 42 | 729,136 | 567,911 | 63.9 |  |
| MCCC 1A08421 | MPZW00000000^﹟^ | 3,858,094 | 30 | 1,235,294 | 373,967 | 63.9 |  |
| MCCC 1A03502^T^ | MPZS00000000^﹟^ | 3,653,331 | 5 | 2,098,934 | 2,098,934 | 64.1 |  |
| MCCC 1A02612^T^ | AQRC00000000^﹩^ | 3,928,443 | 47 | 531,809 | 235,161 | 65.3 |  |
| MCCC 1A00513^T^ | AUNB00000000^﹩^ | 3,822,068 | 105 | 455,218 | 116,979 | 60.3 |  |
| MCCC 1A03957^T^ | JHEH00000000^﹩^ | 4,179,056 | 98 | 325,149 | 109,664 | 62.5 |  |

^§:^ IMG Genome ID from the IMG database.

^﹟^: The genome sequences were determined in this study.

^﹩^: The genome sequences were obtained from the GenBank database.

N50 length is defined as the shortest sequence length at 50% of the genome.

**Table S4** The pairwise MLSA similarities values (%) among 23 strains

| No. | MCCC/Original No. | 1 | 2 | 3 | 4 | 5 | 6 | 7 | 8 | 9 | 10 | 11 | 12 | 13 | 14 | 15 | 16 | 17 | 18 | 19 | 20 | 21 | 22 | 23 |
| --- | --- | --- | --- | --- | --- | --- | --- | --- | --- | --- | --- | --- | --- | --- | --- | --- | --- | --- | --- | --- | --- | --- | --- | --- |
| 1 | MCCC 1A00513^T^ | 100 | 85.0 | 84.8 | 84.8 | 85.0 | 84.8 | 84.9 | 84.8 | 85.2 | 84.9 | 85.5 | 85.5 | 85.5 | 86.4 | 84.8 | 84.8 | 85.7 | 84.9 | 85.0 | 85.5 | 85.1 | 85.1 | 85.1 |
| 2 | MCCC 1A02612^T^ | 85.0 | 100 | 90.1 | 90.1 | 89.7 | 89.8 | 90.0 | 90.1 | 89.9 | 90.1 | 91.2 | 91.2 | 91.2 | 87.7 | 89.7 | 89.8 | 90.9 | 90.0 | 100 | 91.2 | 89.6 | 90.1 | 90.2 |
| 3 | MCCC 1A02765 | 84.8 | 90.1 | 100 | 99.4 | 94.9 | 94.8 | 99.2 | 99.2 | 94.7 | 99.4 | 90.6 | 90.6 | 90.6 | 86.7 | 95.0 | 95.1 | 91.0 | 99.0 | 90.1 | 90.8 | 95.1 | 95.1 | 94.9 |
| 4 | MCCC 1A02808 | 84.8 | 90.1 | 99.4 | 100 | 94.7 | 94.6 | 99.1 | 99.3 | 94.6 | 99.3 | 90.6 | 90.6 | 90.6 | 86.8 | 94.9 | 95.0 | 91.0 | 99.0 | 90.1 | 90.8 | 95.0 | 95.0 | 94.8 |
| 5 | MCCC 1A02813 | 85.0 | 89.7 | 94.9 | 94.7 | 100 | 97.4 | 94.9 | 94.9 | 96.6 | 94.7 | 90.6 | 90.6 | 90.6 | 87.4 | 97.1 | 97.2 | 91.0 | 94.8 | 89.7 | 90.8 | 97.0 | 96.8 | 96.8 |
| 6 | MCCC 1A02837 | 84.8 | 89.8 | 94.8 | 94.6 | 97.4 | 100 | 94.6 | 94.7 | 97.2 | 94.6 | 90.8 | 90.7 | 90.8 | 87.3 | 97.6 | 97.1 | 91.2 | 94.7 | 89.8 | 90.9 | 97.3 | 96.9 | 96.7 |
| 7 | MCCC 1A02857 | 84.9 | 90.0 | 99.2 | 99.1 | 94.9 | 94.6 | 100 | 99.2 | 94.7 | 99.0 | 90.7 | 90.7 | 90.7 | 86.8 | 94.9 | 95.1 | 91.1 | 99.0 | 90.0 | 90.9 | 95.0 | 95.1 | 94.9 |
| 8 | MCCC 1A02959 | 84.8 | 90.1 | 99.2 | 99.3 | 94.9 | 94.7 | 99.2 | 100 | 94.7 | 99.1 | 90.7 | 90.7 | 90.7 | 86.8 | 94.9 | 95.1 | 91.1 | 99.0 | 90.1 | 90.9 | 95.1 | 95.1 | 95.0 |
| 9 | MCCC 1A03188 | 85.2 | 89.9 | 94.7 | 94.6 | 96.6 | 97.2 | 94.7 | 94.7 | 100 | 94.5 | 90.8 | 90.8 | 90.8 | 87.1 | 97.0 | 97.3 | 91.2 | 94.7 | 89.9 | 90.9 | 97.5 | 96.7 | 96.7 |
| 10 | MCCC 1A03190 | 84.9 | 90.1 | 99.4 | 99.3 | 94.7 | 94.6 | 99.0 | 99.1 | 94.5 | 100 | 90.6 | 90.7 | 90.6 | 86.8 | 94.8 | 94.9 | 91.1 | 98.8 | 90.1 | 90.8 | 94.9 | 94.9 | 94.8 |
| 11 | MCCC 1A03502^T^ | 85.5 | 91.2 | 90.6 | 90.6 | 90.6 | 90.8 | 90.7 | 90.7 | 90.8 | 90.6 | 100 | 100 | 100 | 88.0 | 90.9 | 90.9 | 93.9 | 90.7 | 91.3 | 99.1 | 90.8 | 91.1 | 91.0 |
| 12 | MCCC 1A03505 | 85.5 | 91.2 | 90.6 | 90.6 | 90.6 | 90.7 | 90.7 | 90.7 | 90.8 | 90.7 | 100 | 100 | 100 | 88.0 | 90.9 | 90.9 | 93.9 | 90.7 | 91.2 | 99.1 | 90.8 | 91.1 | 91.1 |
| 13 | MCCC 1A03506 | 85.5 | 91.2 | 90.6 | 90.6 | 90.6 | 90.8 | 90.7 | 90.7 | 90.8 | 90.6 | 100 | 100 | 100 | 88.0 | 90.9 | 90.9 | 93.9 | 90.7 | 91.3 | 99.1 | 90.8 | 91.1 | 91.0 |
| 14 | MCCC 1A03957^T^ | 86.4 | 87.7 | 86.7 | 86.8 | 87.4 | 87.3 | 86.8 | 86.8 | 87.1 | 86.8 | 88.0 | 88.0 | 88.0 | 100 | 87.2 | 87.1 | 88.1 | 86.8 | 87.7 | 88.0 | 87.1 | 87.7 | 87.2 |
| 15 | MCCC 1A03973 | 84.8 | 89.7 | 95.0 | 94.9 | 97.1 | 97.6 | 94.9 | 94.9 | 97.0 | 94.8 | 90.9 | 90.9 | 90.9 | 87.2 | 100 | 97.7 | 91.1 | 94.8 | 89.7 | 91.1 | 98.2 | 96.7 | 96.8 |
| 16 | MCCC 1A03974 | 84.8 | 89.8 | 95.1 | 95.0 | 97.2 | 97.1 | 95.1 | 95.1 | 97.3 | 94.9 | 90.9 | 90.9 | 90.9 | 87.1 | 97.7 | 100 | 91.0 | 95.1 | 89.8 | 91.1 | 98.1 | 96.9 | 96.7 |
| 17 | MCCC 1A06460^T^ | 85.7 | 90.9 | 91.0 | 91.0 | 91.0 | 91.2 | 91.1 | 91.1 | 91.2 | 91.1 | 93.9 | 93.9 | 93.9 | 88.1 | 91.1 | 91.0 | 100 | 91.1 | 90.9 | 94.1 | 91.3 | 91.2 | 91.2 |
| 18 | MCCC 1A07302^T^ | 84.9 | 90.0 | 99.0 | 99.0 | 94.8 | 94.7 | 99.0 | 99.0 | 94.7 | 98.8 | 90.7 | 90.7 | 90.7 | 86.8 | 94.8 | 95.1 | 91.1 | 100 | 90.0 | 90.9 | 95.0 | 95.1 | 94.9 |
| 19 | MCCC 1A07323 | 85.0 | 100 | 90.1 | 90.1 | 89.7 | 89.8 | 90.0 | 90.1 | 89.9 | 90.1 | 91.3 | 91.2 | 91.3 | 87.7 | 89.7 | 89.8 | 90.9 | 90.0 | 100 | 91.3 | 89.6 | 90.1 | 90.2 |
| 20 | MCCC 1A08421 | 85.5 | 91.2 | 90.8 | 90.8 | 90.8 | 90.9 | 90.9 | 90.9 | 90.9 | 90.8 | 99.1 | 99.1 | 99.1 | 88.0 | 91.1 | 91.1 | 94.1 | 90.9 | 91.3 | 100 | 91.0 | 91.3 | 91.3 |
| 21 | MCCC 1A10143^T^ | 85.1 | 89.6 | 95.1 | 95.0 | 97.0 | 97.3 | 95.0 | 95.1 | 97.5 | 94.9 | 90.8 | 90.8 | 90.8 | 87.1 | 98.2 | 98.1 | 91.3 | 95.0 | 89.6 | 91.0 | 100 | 96.9 | 96.7 |
| 22 | ES.031 | 85.1 | 90.1 | 95.1 | 95.0 | 96.8 | 96.9 | 95.1 | 95.1 | 96.7 | 94.9 | 91.1 | 91.1 | 91.1 | 87.7 | 96.7 | 96.9 | 91.2 | 95.1 | 90.1 | 91.3 | 96.9 | 100 | 97.0 |
| 23 | ES.032 | 85.1 | 90.2 | 94.9 | 94.8 | 96.8 | 96.7 | 94.9 | 95.0 | 96.7 | 94.8 | 91.0 | 91.1 | 91.0 | 87.2 | 96.8 | 96.7 | 91.2 | 94.9 | 90.2 | 91.3 | 96.7 | 97.0 | 100 |

**Table S5** The pairwise dDDH values (%) at the upper 95% confidence interval among 15 representative strains

| No. | MCCC/Original No. | 1 | 2 | 3 | 4 | 5 | 6 | 7 | 8 | 9 | 10 | 11 | 12 | 13 | 14 | 15 |
| --- | --- | --- | --- | --- | --- | --- | --- | --- | --- | --- | --- | --- | --- | --- | --- | --- |
| 1 | MCCC 1A03973 | 100 | 74.0 | 70.6 | 72.5 | 70.7 | 74.2 | 62.6 | 61.7 | 45.6 | 28.6 | 28.9 | 28.6 | 27.1 | 23.4 | 24.3 |
| 2 | MCCC 1A10143^T^ | 74.0 | 100 | 70.5 | 72.1 | 70.6 | 73.7 | 62.5 | 61.8 | 45.5 | 28.5 | 28.7 | 28.6 | 27.0 | 23.3 | 24.0 |
| 3 | MCCC 1A03974 | 70.6 | 70.5 | 100 | 70.5 | 71.1 | 70.3 | 63.3 | 62.2 | 45.4 | 29.0 | 29.0 | 28.6 | 27.6 | 23.1 | 25.1 |
| 4 | MCCC 1A03188 | 72.5 | 72.1 | 70.5 | 100 | 70.4 | 72.1 | 62.5 | 61.8 | 45.6 | 28.7 | 28.8 | 28.5 | 27.9 | 23.2 | 25.3 |
| 5 | MCCC 1A02813 | 70.7 | 70.6 | 71.1 | 70.4 | 100 | 70.6 | 63.1 | 62.4 | 45.4 | 28.6 | 29.0 | 28.6 | 27.3 | 23.1 | 24.4 |
| 6 | MCCC 1A02837 | 74.2 | 73.7 | 70.3 | 72.1 | 70.6 | 100 | 62.5 | 62.0 | 45.5 | 28.5 | 28.7 | 28.7 | 27.0 | 23.2 | 24.1 |
| 7 | ES.031 | 62.6 | 62.5 | 63.3 | 62.5 | 63.1 | 62.5 | 100 | 65.4 | 45.7 | 28.8 | 29.0 | 28.8 | 27.3 | 23.3 | 24.1 |
| 8 | ES.032 | 61.7 | 61.8 | 62.2 | 61.8 | 62.4 | 62.0 | 65.4 | 100 | 45.1 | 28.9 | 28.9 | 28.5 | 27.1 | 23.3 | 24.4 |
| 9 | MCCC 1A07302^T^ | 45.6 | 45.5 | 45.4 | 45.6 | 45.4 | 45.5 | 45.7 | 45.1 | 100 | 28.1 | 28.2 | 28.0 | 26.6 | 22.9 | 23.9 |
| 10 | MCCC 1A06460^T^ | 28.6 | 28.5 | 29.0 | 28.7 | 28.6 | 28.5 | 28.8 | 28.9 | 28.1 | 100 | 40.7 | 40.8 | 28.1 | 23.4 | 24.3 |
| 11 | MCCC 1A08421 | 28.9 | 28.7 | 29.0 | 28.8 | 29.0 | 28.7 | 29.0 | 28.9 | 28.2 | 40.7 | 100 | 93.0 | 28.3 | 23.3 | 24.3 |
| 12 | MCCC 1A03502^T^ | 28.6 | 28.6 | 28.6 | 28.5 | 28.6 | 28.7 | 28.8 | 28.5 | 28.0 | 40.8 | 93.0 | 100 | 27.9 | 23.3 | 23.9 |
| 13 | MCCC 1A02612^T^ | 27.1 | 27.0 | 27.6 | 27.9 | 27.3 | 27.0 | 27.3 | 27.1 | 26.6 | 28.1 | 28.3 | 27.9 | 100 | 23.0 | 25.0 |
| 14 | MCCC 1A00513^T^ | 23.4 | 23.3 | 23.1 | 23.2 | 23.1 | 23.2 | 23.3 | 23.3 | 22.9 | 23.4 | 23.3 | 23.3 | 23.0 | 100 | 23.9 |
| 15 | MCCC 1A03957^T^ | 24.3 | 24.0 | 25.1 | 25.3 | 24.4 | 24.1 | 24.1 | 24.4 | 23.9 | 24.3 | 24.3 | 23.9 | 25.0 | 23.9 | 100 |

**Table S6** The pairwise ANI values (%) among 15 representative strains

| No. | MCCC/Original No. | 1 | 2 | 3 | 4 | 5 | 6 | 7 | 8 | 9 | 10 | 11 | 12 | 13 | 14 | 15 |
| --- | --- | --- | --- | --- | --- | --- | --- | --- | --- | --- | --- | --- | --- | --- | --- | --- |
| 1 | MCCC 1A03973 | 100 | 96.67 | 96.15 | 96.38 | 96.19 | 96.63 | 94.91 | 94.82 | 91.31 | 83.16 | 82.95 | 83.12 | 81.99 | 78.56 | 77.88 |
| 2 | MCCC 1A10143^T^ | 96.67 | 100 | 96.15 | 96.43 | 96.14 | 96.64 | 94.91 | 94.76 | 91.10 | 82.97 | 83.13 | 83.13 | 81.86 | 78.53 | 77.76 |
| 3 | MCCC 1A03974 | 96.15 | 96.15 | 100 | 96.17 | 96.15 | 96.11 | 95.05 | 94.86 | 91.07 | 83.36 | 83.49 | 83.23 | 82.50 | 79.31 | 77.72 |
| 4 | MCCC 1A03188 | 96.38 | 96.43 | 96.17 | 100 | 96.05 | 96.50 | 94.88 | 94.83 | 91.26 | 83.19 | 83.41 | 83.18 | 82.74 | 79.51 | 77.98 |
| 5 | MCCC 1A02813 | 96.19 | 96.14 | 96.15 | 96.05 | 100 | 96.14 | 94.96 | 94.85 | 91.03 | 82.82 | 83.28 | 83.06 | 81.39 | 78.68 | 77.92 |
| 6 | MCCC 1A02837 | 96.63 | 96.64 | 96.11 | 96.50 | 96.14 | 100 | 94.92 | 94.75 | 91.25 | 83.04 | 82.95 | 83.08 | 81.84 | 78.60 | 77.87 |
| 7 | ES.031 | 94.91 | 94.91 | 95.05 | 94.88 | 94.96 | 94.92 | 100 | 95.44 | 91.36 | 83.45 | 83.30 | 83.20 | 82.12 | 78.58 | 77.89 |
| 8 | ES.032 | 94.82 | 94.76 | 94.86 | 94.83 | 94.85 | 94.75 | 95.44 | 100 | 90.98 | 83.11 | 83.14 | 82.95 | 82.07 | 78.75 | 77.93 |
| 9 | MCCC 1A07302^T^ | 91.31 | 91.10 | 91.07 | 91.26 | 91.03 | 91.25 | 91.36 | 90.98 | 100 | 82.44 | 82.68 | 82.81 | 81.61 | 78.21 | 77.78 |
| 10 | MCCC 1A06460^T^ | 83.16 | 82.97 | 83.36 | 83.19 | 82.82 | 83.04 | 83.45 | 83.11 | 82.44 | 100 | 89.53 | 89.86 | 82.70 | 78.54 | 77.89 |
| 11 | MCCC 1A08421 | 82.95 | 83.13 | 83.49 | 83.41 | 83.28 | 82.95 | 83.30 | 83.14 | 82.68 | 89.53 | 100 | 98.95 | 82.70 | 78.40 | 78.13 |
| 12 | MCCC 1A03502^T^ | 83.12 | 83.13 | 83.23 | 83.18 | 83.06 | 83.08 | 83.20 | 82.95 | 82.81 | 89.86 | 98.95 | 100 | 82.64 | 78.32 | 77.87 |
| 13 | MCCC 1A02612^T^ | 81.99 | 81.86 | 82.50 | 82.74 | 81.39 | 81.84 | 82.12 | 82.07 | 81.61 | 82.70 | 82.70 | 82.64 | 100 | 79.68 | 77.78 |
| 14 | MCCC 1A00513^T^ | 78.56 | 78.53 | 79.31 | 79.51 | 78.68 | 78.60 | 78.58 | 78.75 | 78.21 | 78.54 | 78.40 | 78.32 | 79.68 | 100 | 78.66 |
| 15 | MCCC 1A03957^T^ | 77.88 | 77.76 | 77.72 | 77.98 | 77.92 | 77.87 | 77.89 | 77.93 | 77.78 | 77.89 | 78.13 | 77.87 | 77.78 | 78.66 | 100 |

**Table S7** Cellular fatty acid composition of the two novel strains and five reference strains.

| Fatty acids | 1 | 2 | 3 | 4 | 5 | 6 | 7 |
| --- | --- | --- | --- | --- | --- | --- | --- |
| C_11:0_ | Nd | 0.50 | 0.18 | 0.26 | Nd | Nd | Nd |
| C_14:0_ | 0.20 | Nd | Tr | Tr | Nd | Tr | Nd |
| C_16:0_ | 2.15 | 1.24 | 1.75 | 2.24 | 0.97 | 6.75 | 3.01 |
| C_17:0_ | 0.36 | Nd | 0.30 | 0.52 | 0.17 | 0.15 | 0.20 |
| C_18:0_ | 2.21 | 1.10 | 2.41 | 1.39 | 1.70 | 2.46 | 2.29 |
| C_10:0_ 3OH | 5.33 | 4.39 | 4.31 | 5.64 | 5.07 | Nd | 5.45 |
| C_11:0_ 3OH | 0.19 | Nd | Tr | 0.15 | Tr | 0.13 | 0.15 |
| C_12:0_ 3OH | Nd | Nd | Tr | 0.12 | Nd | 2.19 | 0.25 |
| C_16:0_ 3OH | 0.21 | Tr | 0.15 | 0.22 | 0.34 | 0.49 | 0.15 |
| C_17:1_ *ω*7*c* | 0.46 | 0.54 | 0.24 | Nd | 0.72 | 0.79 | 0.74 |
| C_18:0_ 3OH | 4.40 | Nd | Nd | 3.36 | Nd | 4.13 | 4.35 |
| C_18:1_ *ω*7c 11-methyl | 0.78 | 0.22 | 1.38 | 0.52 | 0.31 | 0.54 | 0.48 |
| C_19:0_ 10-methyl | 1.63 | 2.44 | 1.49 | 1.15 | 2.24 | 1.08 | 1.24 |
| C_19:0_ cyclo *ω*8*c* | 1.34 | 0.34 | 1.78 | 0.88 | 2.39 | 22.6 | 2.93 |
| iso-C_10:0_ | 0.32 | Nd | Nd | Nd | 0.12 | Nd | 0.04 |
| iso-C_11:0_ | 0.29 | 0.33 | Nd | Nd | 0.13 | Tr | 0.18 |
| iso-C_11:0_ 3OH | 0.15 | 0.10 | 0.12 | 0.12 | 0.16 | Nd | 0.20 |
| iso-C_16:0_ | Nd | 0.24 | 0.14 | Nd | Nd | Nd | Nd |
| Summed Feature 2* | Nd | 0.15 | 0.12 | 1.13 | 0.21 | 0.46 | 0.25 |
| Summed Feature 3* | 2.53 | 0.99 | 2.29 | 2.82 | 0.52 | 0.68 | 4.32 |
| Summed Feature 7* | 0.14 | Nd | 0.23 | Tr | Tr | 0.21 | Nd |
| Summed Feature 8* | 77.04 | 87.07 | 82.3 | 78.4 | 84.4 | 56.3 | 73.5 |

Strains: 1, TAW-CT134^T^; 2, 11.10-0-13^T^; 3, MCCC 1A07302^T^; 4, MCCC 1A02612^T^; 5, MCCC 1A06460^T^; 6, MCCC 1A03957^T^; 7, MCCC 1A00513^T^. The data of for the latter five strains were obtained from the published the results by us. Values were percentages of total fatty acids. Tr, trace amount (less than 0.1%). Nd, not detected.

*Summed Features represent groups of two fatty acids which could not be separated by GLC with the MIDI system. Summed Feature 2: C_14:0_ 3OH and/or iso-C_16:1_ I, Summed Feature 3: C_16:1_*ω*7*c* and/or C_16:1_*ω*6*c*; Summed Feature 7: unknown 18.846 and/or C_19:1_*ω*6*c*; Summed Feature 8: C_18:1_*ω*7*c* and/or C_18:1_*ω*6*c*.
