# Supplementary material for: A Multilocus Sequence Analysis Scheme for Phylogeny of Thioclava Bacteria and Proposal of Two Novel Species
Source: Front Microbiol. 2017 Jul 13;8:1321. doi: 10.3389/fmicb.2017.01321 (PMC5508018; doi:10.3389/fmicb.2017.01321)
Supplement: Supplementary file 2 [file DataSheet2.PDF]

GREAT AT SMALL THINGS

BCCM DCG
BCCM IHEM
BCCM ITM
BCCM LMBP
BCCM MUCL
BCCM ULC
BCCM CC

CERTIFICATE OF DEPOSIT

This is to certify that the following microorganism has been deposited into the BCCM/LMG Bacteria Collection and is available to the public without restriction:

LMG number: **LMG 29615**

Speciesname: **Thioclava sp.**

Depositor: **Liu Yang, Third Institute of Oceanography, Key Lab of Marine Biogenetic Resources - State Oceanic Administration**

Depositor no: **TAW-CT134**

Gent, 07 July 2016

ir. Claudine Vereecke
Public Collection Curator
BCCM/LMG Bacteria Collection
