# Supplementary material for: A Multilocus Sequence Analysis Scheme for Phylogeny of Thioclava Bacteria and Proposal of Two Novel Species
Source: Front Microbiol. 2017 Jul 13;8:1321. doi: 10.3389/fmicb.2017.01321 (PMC5508018; doi:10.3389/fmicb.2017.01321)
Supplement: Supplementary file 3 [file DataSheet3.PDF]

GREAT AT SMALL THINGS

BCCM DCG
BCCM IHEM
BCCM ITM
BCCM LMBP
BCCM MUCL
BCCM ULC
BCCM CC

CERTIFICATE OF DEPOSIT

This is to certify that the following microorganism has been deposited into the BCCM/LMG Bacteria Collection and is available to the public without restriction:

LMG number: **LMG 29618**

Speciesname: **Thioclava sp.**

Depositor: **Liu Yang, Third Institute of Oceanography, Key Lab of Marine Biogenetic Resources - State Oceanic Administration**

Depositor no: **11.10-0-13**

Gent, 07 July 2016

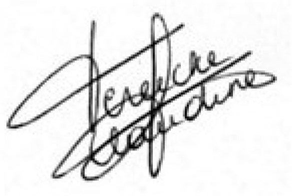

ir. Claudine Vereecke
Public Collection Curator
BCCM/LMG Bacteria Collection

Laboratorium voor Microbiologie - Universiteit Gent (UGent)
K.L. Ledeganckstraat 35 - B-9000 Gent - Belgium
T +32 (0)9 264 51 08 - bccm.lmg@ugent.be

UNIVERSITEIT GENT
Belgian Science Policy Office
.be
