# Supplementary material for: A Multilocus Sequence Analysis Scheme for Phylogeny of Thioclava Bacteria and Proposal of Two Novel Species
Source: Front Microbiol. 2017 Jul 13;8:1321. doi: 10.3389/fmicb.2017.01321 (PMC5508018; doi:10.3389/fmicb.2017.01321)
Supplement: Supplementary file 4 [file DataSheet4.PDF]

国家微生物资源平台
NIMR National Infrastructure of Microbial Resources
MCCC org.cn

中国海洋微生物菌种保藏管理中心
Marine Culture Collection of China

CERTIFICATE OF DEPOSIT

IN MARINE CULTURE COLLECTION OF CHINA

Marine Culture Collection of China
Third Institute of Oceanography, State Oceanic Administration
Daxue Road 178, 361005 Xiamen, Fujian
P. R. China.
Phone/Fax: +86-592-2195177
Email: mccc5177@163.com
Web site: http://www.mccc.org.cn

MCCC 1A03502

***Thioclava* sp. (strain 11.10-0-13) was received for deposit**
in Marine Culture Collection of China from

Jiachuan Pan
Third Institute of Oceanography, State Oceanic Administration
Daxue Road 178, Xiamen 361005, Fujian
P. R. China

on Aug. 23, 2007

and was, after confirming the viability and purity,
allocated the accession number MCCC 1A03502.

The strain is available to any *bona fide* scientific community or individual,
operating in a professional environment
suitable for handling living material of the biohazard group involved.

Xiamen, Apr. 21, 2017

Zongze Shao

Dr. Zongze Shao
Public Collection Curator
Marine Culture Collection of China
